# Appendicitis as a Leading Manifestation of Kawasaki Disease in Older Children

**DOI:** 10.3390/children9020193

**Published:** 2022-02-02

**Authors:** Ya-Ning Huang, Lu-Hang Liu, Jeng-Jung Chen, Yu-Lin Tai, Yih-Cherng Duh, Chien-Yu Lin

**Affiliations:** 1 Department of Pediatrics, MacKay Children’s Hospital, Taipei 10449, Taiwan; yvon1207@gmail.com; 2 Department of Pediatrics, Hsinchu MacKay Memorial Hospital, Hsinchu City 30071, Taiwan; 2081@mmh.org.tw (L.-H.L.); 6134@mmh.org.tw (J.-J.C.); superlof@gmail.com (Y.-L.T.); 3 Department of Biological Science and Technology, National Yang Ming Chiao Tung University, Hsinchu City 30071, Taiwan; 4 Division of Pediatric Surgery, Department of Surgery, Hsinchu MacKay Memorial Hospital, Hsinchu City 30071, Taiwan; 5 MacKay Junior College of Medicine, Nursing and Management, New Taipei City 25245, Taiwan; 6 Department of Medicine, MacKay Medical College, New Taipei City 25160, Taiwan

**Keywords:** Kawasaki disease, appendicitis, intravenous immunoglobulin, acute abdomen, coronary aneurysm

## Abstract

Kawasaki disease (KD) is a systematic inflammatory disease with multiple organ involvement. Timely diagnosis and prompt management are essential for successful treatment. KD, with an atypical presentation, remains a diagnostic challenge for physicians. We report a five-year-old boy who presented with appendicitis. An appendectomy was performed; however, his fever persisted. The boy was diagnosed with KD and intravenous immunoglobulin was administered. His symptoms resolved, and he had an uneventful recovery. Furthermore, we performed a literature review with 13 cases identified in the literature. Most cases were male, and the average age was older than typical for KD. In conclusion, KD may present with abdominal complaints and appendicitis may be a rare initial presentation of KD. Multidisciplinary cooperation and high awareness are warranted for timely diagnosis, especially in older children experiencing persistent fever after an appendectomy.

## 1. Introduction

Kawasaki disease (KD) is a systemic inflammatory disease that predominantly occurs in children less than five years of age. Timely diagnosis and the appropriate treatment are crucial for successful management [1,2]. In the past decade, studies have shown that delayed treatment may raise the risk of a subsequent coronary aneurysm and increase the morbidity and mortality of KD, particularly in infants and older children [3]. The classical manifestations include fever, non-purulent conjunctivitis, cervical lymphadenitis, oral mucosa change, swollen palms or soles, and skin rashes. However, the clinical manifestations are protean and atypical presentations with a delayed diagnosis are not uncommon. KD may present with abdominal complaints as its early cardinal symptoms. In a cohort study of 219 children with KD, Zulian et al. reported that 10 children (4.6%) had severe abdominal complaints as their first presentation. Half of the 219 children developed coronary artery aneurysms despite early immunoglobulin treatment. In addition, atypical KD presentation was accompanied by an acute abdomen in 9 of the 10 children [4]. Atypical manifestations impede physicians from timely diagnosing KD. Furthermore, KD with initial manifestations mimicking appendicitis has been reported. Recognizing KD with atypical presentations earlier remains a challenge for physicians.

## 2. Case Report

**Figure 1:**
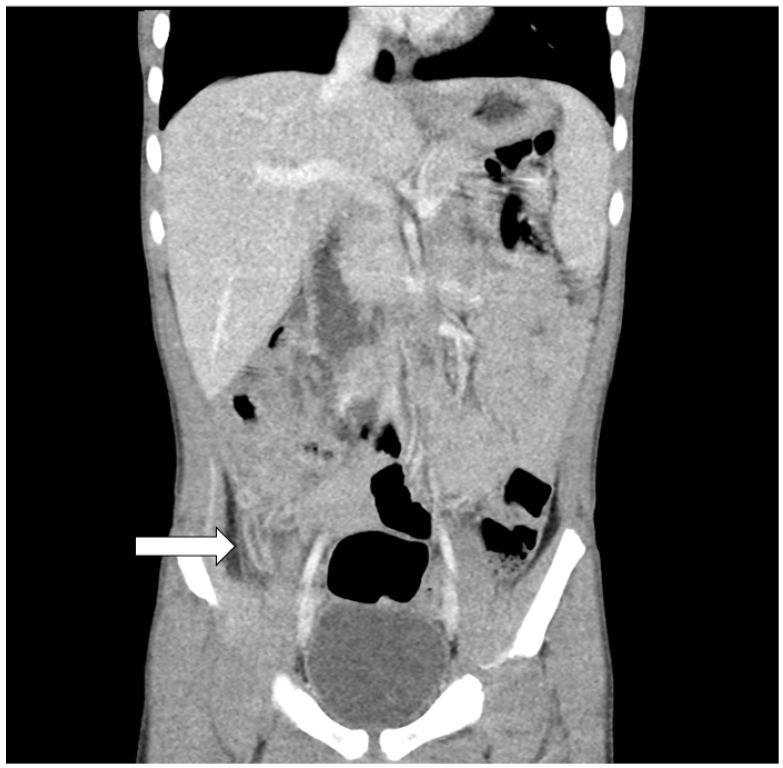
Computed tomography of abdomen demonstrated appendicitis (arrow).

A previously healthy five-year-old boy presented with spiking fevers for 3 days, accompanied by lower abdominal pain that worsened with walking. He had soft stool passage two to three times for 2 days. He had no conjunctivitis, vomiting, chills, bloody diarrhea, rash, or purpura in his legs, or conscious disturbance. Within the day prior to his arrival, peritonitis signs were presented at a local clinic. He was immediately transferred to our emergency department after an acute abdomen was suspected. A plain radiograph showed no perforation or obstruction of the bowels. The number of leukocytes in the blood was 10,300/μL with neutrophile predominant (83%), and the level of C-reactive protein was 11.78 mg/dL. CT scans of the whole abdomen revealed an engorged appendix with wall enhancement, adjacent cecal wall thickening, and perifocal fat stranding over the right lower abdomen, suggesting acute appendicitis (Figure 1). A laparoscopic appendectomy was then performed, and the inflamed hyperemic and engorged appendix was completely resected. Pathological examination showed moderate infiltration of lymphocytes and neutrophils in a large portion of the serosal, muscular layer of the appendix, which is compatible with acute suppurative appendicitis. After surgery, he still suffered spiking fevers between 39 and 40 °C and was refractory to antibiotics, even with his improved wound condition. An abdominal ultrasound showed no apparent residual abscess or ascites formation. In the 2 days following surgery, itchy polymorphous urticaria developed in his four limbs. An allergic reaction to antibiotics was suspected, and an antihistamine was administered for relief. Bilateral bulbar conjunctival injection, cracked lips, peripheral edema involving four limbs, and a truncal polymorphous rash were noted 5 days after surgery. KD was suspected as the cause. Furthermore, echocardiography was performed within normal limits. Elevated white cell counts (14,100/μL), an erythrocyte sedimentation rate (47 mm/h), and C-reactive protein (4.09 mg/dL) without elevation of transaminases were notable on repeated blood tests. A diagnosis of KD was decided within 8 d of spiking fever. Hence, immunoglobulin (2 g/kg) and a medium dose of aspirin (30 mg/kg in 1 day) were administered immediately on the eighth day from the disease onset. After 8 days of the aforementioned treatment, the child’s fever and other symptoms were resolved without further cardiac complications. Normal white cell count and C-reactive protein was also restored. Desquamation of the fingers and palms developed after 2 weeks of the disease. The child recovered completely and followed up with echocardiography monitoring.

## 3. Discussion and Literature Review

KD may present with abdominal complaints, including appendicitis. The entire underpinning mechanism of KD is not completely elucidated. Several possible mechanisms may explain the systemic inflammation induced by cytokines, including serum interleukin (IL)-6, IL-8, tumor necrosis factor (TNF)-alpha, and serum vascular endothelial growth factor. A systemic inflammatory cascade may cause vascular hyperpermeability, vasculitis and an overall picture of abdominal inflammation and appendicitis [5,6]. Garnett et al. claimed that KD presents itself in many ways and masquerades as other diseases, such as acute abdomen, mesenteric adenitis, intestinal stricture, and gallbladder hydrops, especially in the early stage of the disease [3,7]. Abdominal pain with prolonged fever and high inflammatory biomarkers should be evaluated from a multidisciplinary pediatric team and the possibility of Kawasaki disease should be considered, ideally before the surgery. The importance of multidisciplinary cooperation and high awareness of KD in patients with abdominal complains are reinforced.

Acute appendicitis may be a rare initial presentation of KD and we performed a literature review of KD with initial presentations of appendicitis (Table 1). Several case reports and one case series from 1998 to 2021 proposed that KD mimics acute appendicitis [3,4,6,7,8,9,10,11]. Since 1998, the 12 cases from those studies were identified and the majority were male (8/9). Appendicitis is a leading manifestation of KD more common in older children between three and seven years of age. Acute appendicitis is an unusual cause of fever with abdominal pain for children at those ages [9]. Of the 12 children who first presented with fever and abdominal pain, all (12/12, 100%) had peritonitis and received an appendectomy, whereas 75% (9/12) were diagnosed with appendicitis by histopathologic reports; 17% (2/12) were diagnosed with mesenteric adenitis, not appendicular vasculitis, and 33% (4/12) had cardiac involvement with coronary artery dilatation or aneurysms as their first presentation of KD. They all presented with acute abdomen before the typical signs of KD, elevated white cell count and C-reactive protein. They also had persistent fever and conjunctivitis. Some of the children had maculopapular rash (10/12), cracked lips (7/12), desquamation of the fingers (5/12), and edematous change to the extremities (5/12). In our case, acute appendicitis was the first presented manifestation of KD, as mentioned by previous case reports. Since gastrointestinal symptoms often precede the classical manifestation of KD, accurate diagnosis is easily overlooked. High awareness and keen judgment of the clinician diagnosing KD are crucial in treating patients with appendicitis and persistent fever after an appendectomy, especially for younger children with appendicitis.

Mevalonate kinase deficiency (MKD) is a rare genetic disorder characterized with periodic fever. A variety of symptoms may be associated with MKD, including abdominal complaints, lymphadenopathy, skin lesions, and arthralgia. Diagnosis of MKD can be made by identifying mutations in the *mevalonate kinase* (MVK) gene and detecting increased excretion of mevalonic acid in the urine. However, diagnosis is often delayed and the initial manifestations may be indistinguishable from incomplete KD [12]. Diagnostic testing of MKD should be considered in KD patients with recurrent fever. Our patient had no more fever episodes after treatment and underwent an uneventful recovery at the half-year follow-up. He was a Han Chinese and no family history or consanguinity of genetic disorders was reported. His intelligence, growth, and development are normal. MKD was not suspected.

The emerging coronavirus disease 2019 (COVID-19) has caused enormous crises worldwide [13,14]. Multisystem Inflammation Syndrome in Children (MIS-C) is a newly occurring disease caused by severe acute respiratory syndrome coronavirus 2 (SARS-CoV-2) infection, mimicking KD. Gastrointestinal involvement is common in children with MIS-C, and approximately two-thirds are older than five years of age [15]. MIS-C with acute appendicitis was recently reported by Jackson et al. during the COVID-19 pandemic era [16]. Thus, the clinical presentation of these two diseases shows similarities, and a careful, differential approach is warranted. The polymerase chain reaction of our patient for SARS-CoV-2 was negative, and no contact or travel history was associated. MIS-C has not been reported in Taiwan at present, and our case reminds physicians about the possible manifestation of KD in the COVID-19 era.

## 4. Conclusions

KD may present with abdominal complaints and appendicitis may be a rare initial presentation of KD. Appendicitis precedes other typical features as a leading manifestation of KD, which impedes timely diagnosis and proper treatment within ten days. For children who present recalcitrant fever accompanied by acute abdomen, aggressive clinical suspicion of Kawasaki disease is necessary, especially with elevated white cell counts and C-reactive protein. The early detection and diagnosis of Kawasaki disease help prevent further cardiac complications in children.

## Figures and Tables

**Table 1:** Clinical characteristics of Kawasaki Disease mimicking appendicitis in 13 children.

Reference	Demographic Profile	Clinical Presentation					Treatment		Outcome
Study (Publication Year)	AGE (Years Old), GENDER	Completed KD	WBC (10^3^/uL) /CRP (mg/dL)	Sonography	Computed Tomography	Histological Findings	Surgery	IVIG	Coronary Artery Aneurysms
Our patient Chiba et al. (1998) [7] Zulian et al. (2003) [4] Ulloa-Gutierrez et al. (2004) [8] Bartlett et al. (2006) [10] Maggio et al. (2007) [9] Miyamoto et al. (2013) [6] Garnett et al. (2014) [3] Velez-Tirado et al. (2019) [11]	5, M 6, M 4.6, M 5.4, M 4, M 7, M 3, M 5, M 3, M 7, F Three cases	+ + + + + + + - + + 3/3 +	10.3/11.8 21.3/11 12.6/7 21.6/6.9 24.2/14.9 23.2/16.7 Both elevated 15.9/15.5 Both elevated Both elevated NA	- NA - Appendicitis - - Appendicitis NA - NA NA	Appendicitis NA NA NA NA Post-operation NA Appendicitis Pelvic ascites All pelvic ascites 1/3 Perforation 2/3 Appendicitis	Appendicular vasculitis Acute phlegmonous appendicitis Inflammatory change with edema Acute transmural inflammation, arteritis Mesenteric adenitis NA Appendicular vasculitis Mesenteric adenitis Acute appendicitis Perforated suppurative appendicitis 2/3: Appendicitis 1/3: Serositis	+ + + + + + + + + + All +	+ + + + + + + + + + All +	Normal Normal Normal Normal Fusiform LCA Normal Saccular form -, pericardial effusion +, follow up normal LCA, persisted aneurysm All Normal

Abbreviations: +, present; -, absent; 1/3, one third of cases; 2/3: two thirds of cases; f/u, follow up; IVIG: intravenous immunoglobulin; KD, Kawasaki disease; LCA, left coronary aneurysm; NA, not available; PE, pericardial effusion.

## Data Availability

Not applicable.

## References

[1] Rife E., Gedalia A. (2020). Kawasaki disease: An update. Curr. Rheumatol. Rep..

[2] Yang Y.L., Kuo H.C. (2021). Public health interventions for COVID-19 reduce kawasaki disease in Taiwan. Children.

[3] Garnett G.M., Kimball S., Melish M.E., Thompson K.S., Puapong D.P., Johnson S.M., Woo R.K. (2014). Appendicitis as the presenting manifestation of kawasaki disease. Pediatr. Surg. Int..

[4] Zulian F., Falcini F., Zancan L., Martini G., Secchieri S., Luzzatto C., Zacchello F. (2003). Acute surgical abdomen as presenting manifestation of kawasaki disease. J. Pediatr..

[5] Yasukawa K., Terai M., Shulman S.T., Toyozaki T., Yajima S., Kohno Y., Rowley A.H. (2002). Systemic production of vascular endothelial growth factor and fms-like tyrosine kinase-1 receptor in acute kawasaki disease. Circulation.

[6] Miyamoto K., Yamazaki Y., Okamoto K., Tsuboi T., Hirao J., Arisaka O. (2013). Kawasaki disease: Relationship between acute surgical abdomen and cytokine profiles. Pediatr. Infect. Dis. J..

[7] Chiba T. (1998). Two cases of appendicitis in kawasaki disease. Nihon Geka Hokan Arch. Fur Jpn. Chir..

[8] Ulloa-Gutierrez R., Gutierrez-Alvarez R., Avila-Aguero M.L. (2004). Kawasaki disease mimicking an acute appendicitis. J. Pediatr..

[9] Maggio M.C., Liotta A., Vitaliti S.M., Corsello G. (2007). A case of kawasaki disease mimicking acute appendicitis. Med. J. Aust..

[10] Bartlett A.H., Dishop M.K., Baker C.J. (2006). An unusual cause of appendicitis in a child. Semin. Pediatr. Infect. Dis..

[11] Velez-Tirado N., Ridaura-Sanz C., Venegas-Montoya E., Scheffler-Mendoza S., Camacho-Moreno R., Otero-Mendoza F., Medina-Vega F.A., Garrido-García L.M., Rivas-Larrauri F., Nakashimada M.A.Y. (2019). Acute abdomen in kawasaki disease. Indian J. Pediatr..

[12] Thors V.S., Vastert S.J., Wulffraat N., van Royen A., Frenkel J., de Sain-van der Velden M., de Koning T.J. (2014). Periodic fever in mvk deficiency: A patient initially diagnosed with incomplete kawasaki disease. Pediatrics.

[13] Chiu N.-C., Chi H., Tu Y.-K., Huang Y.-N., Tai Y.-L., Weng S.-L., Chang L., Huang D.T.-N., Huang F.-Y., Lin C.-Y. (2021). To mix or not to mix? A rapid systematic review of heterologous prime–boost COVID-19 vaccination. Expert Rev. Vaccines.

[14] Lien C.H., Lee M.D., Weng S.L., Lin C.H., Liu L.Y., Tai Y.L., Lei W.T., Liu J.M., Huang Y.N., Chi H. (2021). Repurposing colchicine in treating patients with COVID-19: A systematic review and meta-analysis. Life.

[15] Dufort E.M., Koumans E.H., Chow E.J., Rosenthal E.M., Muse A., Rowlands J., Barranco M.A., Maxted A.M., Rosenberg E.S., Easton D. (2020). Multisystem inflammatory syndrome in children in new york state. N. Engl. J. Med..

[16] Jackson R.J., Chavarria H.D., Hacking S.M. (2020). A case of multisystem inflammatory syndrome in children mimicking acute appendicitis in a COVID-19 pandemic area. Cureus.

